# Relationship between Regulatory T Cells and Immune Activation in Human Immunodeficiency Virus-Infected Patients Interrupting Antiretroviral Therapy

**DOI:** 10.1371/journal.pone.0011659

**Published:** 2010-07-21

**Authors:** Laurence Weiss, Christophe Piketty, Lambert Assoumou, Céline Didier, Laure Caccavelli, Vladimira Donkova-Petrini, Yves Levy, Pierre-Marie Girard, Marianne Burgard, Jean-Paul Viard, Christine Rouzioux, Dominique Costagliola, the ANRS 116 SALTO study group

**Affiliations:** 1 Faculté de Médecine, Université Paris Descartes, Paris, France; 2 Assistance Publique - Hôpitaux de Paris (AP-HP), Hôpital Européen Georges Pompidou, Paris, France; 3 Unité “Régulation des Infections Rétrovirales”, Institut Pasteur, Paris, France; 4 INSERM U943, Paris, France; 5 Université Pierre et Marie Curie - Paris 6, UMR S 943, Paris, France; 6 INSERM, Unité U841, Université Paris XII, Créteil, France; 7 AP-HP, Groupe Henri-Mondor Albert-Chenevier, Créteil, France; 8 AP-HP, Hôpital Saint-Antoine, Paris, France; 9 AP-HP, Hôpital Necker Paris, France; Institut Pasteur, France

## Abstract

Persistent immune activation plays a central role in driving Human Immunodeficiency Virus (HIV) disease progression. Whether CD4+CD25+ regulatory T cells (Tregs) are harmful by suppressing HIV-specific immune responses and/or beneficial through a decrease in immune activation remains debatable. We analysed the relationship between proportion and number of regulatory T cells (Tregs) and immune activation in HIV-infected patients interrupting an effective antiretroviral therapy (ART). Twenty-five patients were included in a substudy of a prospective multicenter trial of treatment interruption (TI) (ANRS 116). Proportions and numbers of Tregs and the proportion of activated CD4 and CD8 T cells were assessed at baseline and month 12 (M12) of TI. Specific anti-HIV CD4 and CD8 responses were investigated at baseline and M12. Non parametric univariate analyses and multivariate linear regression models were conducted. At baseline, the proportion of Tregs negatively correlated with the proportion of HLA-DR^+^CD8^+^T cells (r = −0.519). Following TI, the proportion of Tregs increased from 6.3% to 7.2% (p = 0.029); absolute numbers of Tregs decreased. The increase in the proportion of HLA-DR^+^CD38^+^CD8^+^T cells was significantly related to the increase in proportion of Tregs (p = 0.031). At M12, the proportion of Tregs did not negatively correlate with CD8 T-cell activation. Nevertheless, Tregs retain a suppressive function since depletion of Treg-containing CD4^+^CD25^+^ cells led to an increase in lymphoproliferative responses in most patients studied. Our data suggest that Tregs are efficient in controlling residual immune activation in patients with ART-mediated viral suppression. However, the insufficient increase in the proportion and/or the decrease in the absolute number of Tregs result in a failure to control immune activation following TI.

**Trial Registration:**

ClinicalTrials.gov NCT00118677

## Introduction

HIV infection is associated with a progressive depletion of CD4^+^ T lymphocytes and defective HIV specific T-cell responses. Persistent immune activation plays a central role in driving CD4 T cell depletion and progression to AIDS [1], [2], [3].

Regulatory T cells (Tregs) may influence the outcome of various infections [4]. CD4^+^CD25^+^Treg cells are able to suppress antigen-specific T-cell responses against a variety of pathogens and also to control inappropriate or exaggerated immune activation induced by various pathogens, thus limiting immune-mediated tissue damage [5], [6], [7]. In HIV/SIV infection, Tregs, capable of suppressing HIV/SIV-specific immune responses, are detected in peripheral blood and lymphoid tissues and may contribute to immune suppression [8], [9], [10], [11], [12], [13]. Whether these cells are harmful by suppressing HIV-specific immune responses or beneficial through a decrease in immune activation remains debatable. Conflicting data have been reported regarding the relationship between Treg activity, immune activation and HIV/SIV disease progression. The level of Tregs has been found to be unaffected, expanded or decreased with disease progression [9], [14], [15], [16], [17], [18]. Similarly, results regarding the ability of Tregs to control chronic immune activation associated with HIV/SIV infection were not consistent among studies [19], [20], [21], [22], [23]. These discrepancies may result from a disparity in the markers used to identify Tregs, the compartments studied (i.e. peripheral blood vs lymphoid tissues) and the stage of the disease. Tregs have been shown to display low surface expression of CD127, irrespective of their level of CD25 expression [24], [25], [26]. Sorted CD4^+^CD25^+^CD127^low/−^T cells exhibit higher levels of intracellular FOXP3 and CTLA-4 and are suppressive in functional assays [27]. In addition, Lim et al [22] recently validated the use of CD4+CD25^+^CD127^low/−^ as a phenotypic marker of CD4 Treg cells in antiretroviral naïve HIV-infected patients.

To date, the effects of viral rebounds on the proportion, number and function of Tregs in patients interrupting combination antiretroviral therapy (cART) are unknown. The level of immune activation following cART discontinuation may hamper the discrimination between activated and Treg cells. We postulated that, following cART interruption, CD4^+^CD25^+^Treg cells might be expanded in the periphery as a consequence of repeated antigen exposure and/or immune activation. We asked the question whether expanded regulatory T cells might be able to control immune activation and therefore influence the immunovirologic outcome after cART interruption. The aim of the study was to analyse the relationship between the proportion and number of Tregs (defined here according to expression of both CD25 and CD127) and immune activation in patients interrupting an effective antiretroviral regimen.

## Methods

The protocol for this trial and supporting CONSORT checklist are available as supporting information; see Checklist S1 and Protocol S1."

### Patients - Study design

The ANRS 116 trial was a prospective, open-label, multicentre trial of ART interruption in patients who had started treatment early (ClinicalTrials.gov Identifier, NCT00118677). The results of this non randomized trial are described elsewhere in the main publication of the study [47]. Briefly, inclusion criteria were as follows: patients treated at CD4 cell count >350/µL and plasma viral load (VL)<50,000 copies/ml, receiving stable ART within 6 months prior to inclusion with no more than one prior treatment failure and exhibiting, at baseline, CD4 cell count >450/µL and stable VL <5000 copies/mL. All patients provided written informed consent; the study was approved by the ethical committee of Hôpital Pitié-Salpétrière, Paris, France. Antiretrovirals were interrupted at Day 0 (Baseline). Criteria to resume ART were a decrease in CD4 cell count to below 300/µL or the occurrence of a stage B or C event [28]. Patient disposition during the ANRS 116 trial is presented in Flowchart S1.

The Tregs'study was proposed to all patients from the Paris area sites. Peripheral blood mononuclear cells (PBMCs), were collected at baseline (before TI) and after 12 months of TI (M12). PBMCs were used immediately for proliferation assays and cryopreserved for phenotypic studies and ELISpot assays.

An immunological substudy was systematically proposed to patients enrolled in the main study in centers from Paris area. Twenty-five patients (18 men and 7 women) were enrolled in the substudy. Twelve patients were receiving a non-nucleoside reverse transcriptase inhibitor-based regimen; 3 patients a protease inhibitor-based regimen; 8 patients a triple nucleoside analogue therapy while 2 patients received a dual nucleoside analogue therapy.

### Flow Cytometric Analysis

The following antibodies were used: anti-CD4 conjugated to phycoerythrin(PE)-Cy7 or Texas Red-PE (ECD), anti-CD8 PC5, anti-CD127 conjugated to fluorescein isothiocyanate (FITC), anti-CD25 PE, anti-CD3 conjugated to allophycocyanin (APC), anti-HLA-DR FITC, anti-CD38 PE (Beckman Coulter). Cells from healthy controls were used in all staining experiments. Analyses were performed using FC500 flow cytometer and CXP software (Beckman Coulter) on at least 1000 gated events.

### Proliferation and suppressive assays

As previously described [8], freshly-obtained PBMCs were assessed for proliferative capacities following stimulation with HIV-p24 protein (Protein Science Corp.), purified tuberculin (PPD, Statens Serum Institute) and cytomegalovirus (CMV) antigen (Bio Whittaker). Stimulation indexes were calculated by dividing the mean cpm of stimulated cells by the mean cpm of unstimulated cells. A stimulation index ≥3 and ≥3000 cpm incorporated was considered as a positive response.

We tested the suppressive activity of CD4^+^CD25^+^Tregs in 10 patients for whom fresh samples were available at M12 of TI. Purified CD4+ T lymphocytes (RosetteSep™ CD4+ enrichment antibody cocktail, StemCell Technologies) were incubated with CD25^+^ magnetic beads (Myltenyi Biotec) for 30 min at 4°C. The CD25^−^ fraction (>90% purity) was collected. Unfractionated CD4^+^ cells and CD4^+^CD25^−^ cells cultured in the presence of 1×10^4^ autologous monocytes were assessed for proliferative capacities.

### ELISpot assays

ELISpot assays were performed as previously described [29]. A set of 18 pools of 15-mer synthetic HIV peptides covering Gag, Reverse Transcriptase and Nef regions, synthesized by Neosystem and kindly supplied by the ANRS was used. Briefly, 96-well polyvinylidene difluoride plates (Millipore, Molsheim, France) were coated with capture antihuman IFN-γ mAb at 1 µg/ml (Diaclone, Besançon, France). PBMC were added in triplicate wells at 1×10^5^ cells per well in the presence of pools of HIV peptides (final concentration of 2 µg/ml) or the combination of phorbol 12-myristate 13-acetate, PMA: 100 ng/mL) (Sigma, St Louis,MO) and ionomycin (10 mM) (Sigma) as positive controls or in the presence of culture medium as negative controls. After 20 h incubation and washing, the second biotinylated mouse anti-IFN-γ monoclonal antibody (Diaclone) was added, followed by streptavidin-alkaline phosphatase conjugate (Diaclone) and chromogen substrate (nitroblue tetrazolium/5-bromo-4-chloro-3-indolylphosphate toluidinemix from Diaclone). Frequencies of antigen-specific spot-forming cells (SFC) were measured with an automated microscope (Zeiss).Results were expressed as SFC per million PBMC and were calculated for each pool of peptides as follows: 10× (mean SFC/10^5^ cells from three antigen-stimulated wells - mean SFC/10^5^ cells from unstimulated wells).

### Virological assays

Plasma HIV-RNA levels were determined in each study site, using the locally available technique with a lower limit of detection of 400 copies/mL. HIV-DNA levels in PBMCs were measured by real time PCR, as described elsewhere [30]. Final results were expressed as the log_10_ number of HIV-1 DNA copies per 10^6^ PBMCs (threshold: 60 copies/10^6^ PBMCs).

### Statistical analyses

Non parametric tests were used to avoid the impact of potential outlier values in a small study. The changes between baseline and M12 after TI were compared using paired Wilcoxon test for continuous variables and McNemar test for categorical variables. The association between markers of interest at baseline, at M12, association between markers at baseline and the change between baseline and M12, were tested using Spearman's non parametric correlation to study association of two continuous variables and Mann-Whitney test to study association of one continuous variable and one categorical variable. Multivariable linear regressions were used to assess the factors independently associated with the proportion of Tregs at baseline and the change in the proportion of Tregs including variables with p-value <0.20 in the univariate analyses. For the HIV-RNA levels at M12, the change in HIV-DNA and the change in CD4 count between baseline and M12, we systematically assessed, in multivariable linear regressions, the role of baseline values of CD4 counts, HIV-DNA levels, CD4 and CD8 T-cell activation and the baseline proportion of Tregs. A backward elimination technique was used to determine the independent factors associated with each endpoint, limiting the number of variables left in the model because of the small study. All reported p values are two-tailed, with a significance level of 0.05. We used FDR method to account for multiplicity issue and we used corrected p value from SAS 9.1. We have also checked linear model assumptions before their used. Analyses were performed with the SPSS software package version 15.0 for Windows (SPSS Inc.) and with the SAS software package version 9.1 for Windows (Cary NC, USA).

## Results

### Study population

The patients' characteristics are depicted in Table 1. The patients did not differ from the overall study population in terms of age, sex, CD4 counts, HIV-RNA and HIV-DNA levels. All patients had plasma HIV-RNA levels <400 copies at baseline. In patients with cART-mediated suppression, HIV-DNA level in PBMCs was the unique virologic marker available. None of the 25 patients had resumed cART at M12.

**Table 1:** Patients' characteristics.

N = 25		Median (IQR$)	P value* M12 vs BL
Gender (male), n (%)		18 (72%)	
Age (years), median (IQR)		41 (37–47)	
Transmission group, n (%)	Homo/bisexual men	14 (56%)	
	Heterosexuals	8 (32%)	
	Intravenous drug users	2 (8%)	
Duration of cART (years), median (IQR)		5 (3–6)	
CD4 cells count (cells/mm^3^), median (IQR)			
	Baseline**	816 (624–892)	P<0.001, n = 25
	Month 12	497 (381–618)	
Plasma HIV-RNA (log_10_ copies/mL), median (IQR),			
	Baseline**	<2.6	P<0.001, n = 25
	Month 12	4.25 (3.69–4.57)	
HIV DNA (log_10_ copies/10^6^ PBMCs), median (IQR)			
	Baseline**	2.56 (2.00–2.93)	P<0.001, n = 25
	Month 12	3.13 (2.67–3.49)	
%CD4+CD25+CD127−/low/CD4+			
	Baseline**	6.3 (4.4–7.8)	P = 0.038, n = 25
	Month 12	7.2 (4.7–10.8)	
Absolute values CD4+CD25+CD127−/low (/µL)			
	Baseline**	41.9 (33.1–61.2)	P = 0.010, n = 25
	Month 12	36.4 (26.1–46.3)	
% HLA-DR+CD38+/CD8+ T cells			
	Baseline**	0.4 (0.2–0.6)	P = 0.019, n = 25
	Month 12	0.5 (0.2–1.7)	
% CD38+/CD8+ T cells			
	Baseline**	8.8 (5.8–11.1)	P = 0.004, n = 25
	Month 12	11.9 (7.7–17.3)	
% HLA-DR+/CD8+ T cells			
	Baseline**	3.2 (1.7–5.0)	P = 0.904, n = 25
	Month 12	3.6 (1.5–5.4)	
%CD38+/CD4+			
	Baseline**	10.5 (8.9–17.1)	P<0.001, n = 25
	Month 12	23.6 (18.2–32.3)	
%HLA-DR+/CD4+			
	Baseline**	3.4 (2.0–4.4)	P = 0.794, n = 25
	Month 12	2.6 (1.8–4.6)	
Anti-HIV CD8 T-cell responses			
Total SFC/10^6^ PBMC to HIV pools, n = 21			
	Baseline**	485 (154–1397)	P = 0.006, n = 21
	Month 12	2598 (1053–3810)	
Number of positive HIV pools, n = 21			
	Baseline**	2 (1–5)	P = 0.023, n = 21
	Month 12	4 (3–7)	
Anti-HIV CD4 T-cell responses			
p24-LPR, % responders, n = 22	Baseline**	6 (27.3%)	P = 0.068, n = 22
	Month 12	1 (4.5%)	

$ IQR: Interquartile Range.

*Paired Wilcoxon test for continuous variables and Mc Nemar test for categorical variables.

**Baseline (BL)  =  before treatment interruption.

At baseline, HIV-DNA levels in PBMCs negatively correlated with CD4 T-cell counts (n = 25, r = −0.430; p = 0.039), anti-HIV-CD8 T-cell responses (n = 22, r = −0.502; p = 0.026) and anti-p24 CD4 T-cell proliferation (n = 22, r = −0.425; p = 0.055).

At M12 of TI, HIV-RNA and HIV-DNA levels strongly correlated (n = 25, r = 0.852; p<0.001). CD4 cell counts negatively correlated with viral load (n = 25, r = −0.572; p = 0.009 for plasma HIV-RNA levels and r = −0.421; p = 0.042 for HIV-DNA levels). As expected [31], specific anti-HIV CD8 T-cell responses were amplified and broadened. Among the 22 patients with available data for anti-HIV-p24 lymphoproliferative CD4 responses at baseline and month 12, the proportion of responses decreased from 27% to 4%.

### In patients with cART-mediated viral suppression, the proportion of regulatory T cells negatively correlated with the proportion of CD8^+^ T cells expressing HLA-DR

**Figure 1:**
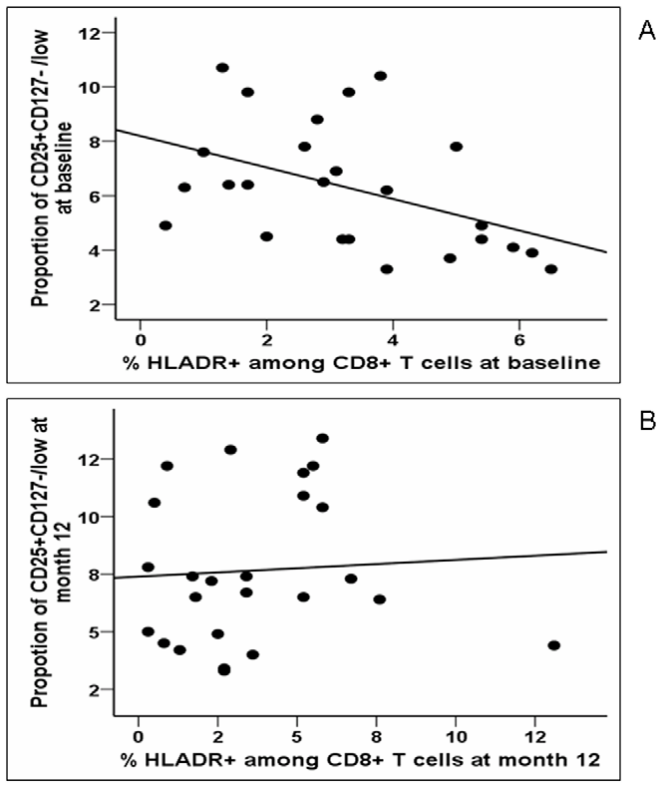
The proportion of Tregs negatively correlated with CD8 T-cell activation at baseline but not at 12 months of treatment interruption (TI). Panel A: Relationship between the percentages of CD8+ T cells expressing HLA-DR (x-axis) and the proportion of CD25+ CD127−/low Tregs among CD4+ T cells at baseline prior to TI (y-axis). Spearman's correlation coefficient R = −0.519, p = 0.008 Panel B: Relationship between the percentages of CD8+ T cells expressing HLA-DR and the proportion of CD25+ CD127−/low Tregs among CD4+ T cells after 12 months of TI (y-axis). Spearman's correlation coefficient R = 0.129; p = 0.539.

At baseline, a median of 6.3% of CD4^+^ T cells expressed the CD25^+^CD127^−/low^ phenotype, defined here as Tregs. To investigate the role of Tregs in controlling immune activation in vivo, we sought a relationship between the proportion and/or the numbers of Tregs and the level of T-cell activation. CD4 and CD8 T-cell activation were linked since the proportion of CD8^+^ T cells HLA-DR^+^ positively correlated with the proportion of CD4^+^ T cells HLA-DR^+^ (n = 25, r = 0.525; p = 0.014). As illustrated in Fig. 1a, the proportion of Tregs at baseline negatively correlated with the percentage of activated HLA-DR^+^ CD8^+^T cells (n = 25, r = −0.519; p = 0.015). Interestingly, the proportion of Tregs positively correlated with HIV-DNA in PBMCs (n = 25, r = 0.553; p = 0.010) and negatively correlated with anti-HIV CD8 T-cell responses (n = 22, r = −0.461; p = 0.039).

In the multivariable analysis, only the proportion of CD8^+^T cells expressing HLA-DR was found to be independently associated with the proportion of Tregs at baseline (n = 22, β = −0.654±0.225, p = 0.009and β = −2.187±0.878, p = 0.022, respectively).

### Following cART interruption, the proportion of Tregs increased as a result of immune activation

**Figure 2:**
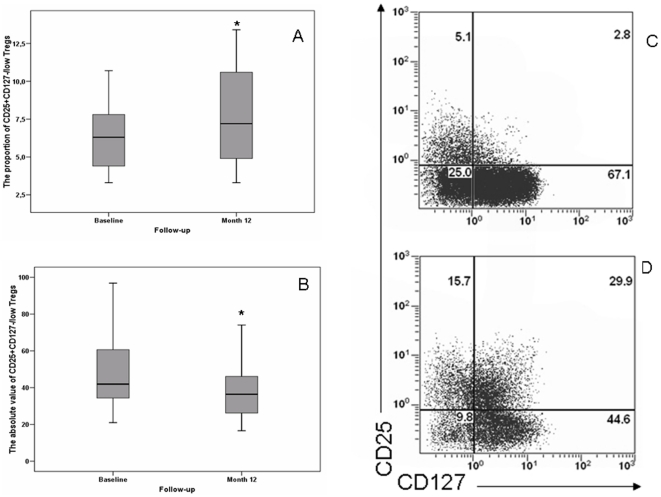
Proportion and absolute numbers of CD4+CD25+CD127−/low Tregs at baseline prior to treatment interruption (TI) and after 12 months off-treatment. The expression of CD25 and CD127 was determined on thawed cryopreserved PBMC by 5-color flow cytometry after successively combining the FSC/SSC, CD3+ and CD4+ gates. Regulatory T cells were defined as CD25+ and CD127−/low.Results are expressed as percentages (panel A) and absolute numbers (panel B) of CD25+ CD127−/low Tregs among CD4+ T cells at baseline and after 12 months of TI (n = 25). The band near the middle of the box indicates the median, the bottom and the top of the boxes are the 25th and 75th percentile. Asterisks indicate a significant difference between M12 and baseline values. Representative stainings of PBMC from one patient at baseline (panel C) and at 12 months of TI (panel D) are shown.

After 12 months of TI, the proportion of Tregs among CD4^+^ T cells increased from 6.3% to 7.2% (n = 25, p = 0.038). In contrast, absolute numbers of Treg cells decreased according to the drop of CD4 T-cell counts (Table 1, Fig. 2).

Both, the proportion of Tregs and the proportion of CD4^+^ and CD8^+^T cells expressing CD38 positively correlated with plasma HIV-RNA levels at M12 of TI (n = 25, r = 0.470; p = 0.026 for Tregs and r = 0.750; p<0.001 and r = 0.555; p = 0.010 for CD4 and CD8 T-cell activation, respectively). We did not find any relationship between the absolute numbers of Tregs and the level of CD4 or CD8 T-cell activation.

**Figure 3:**
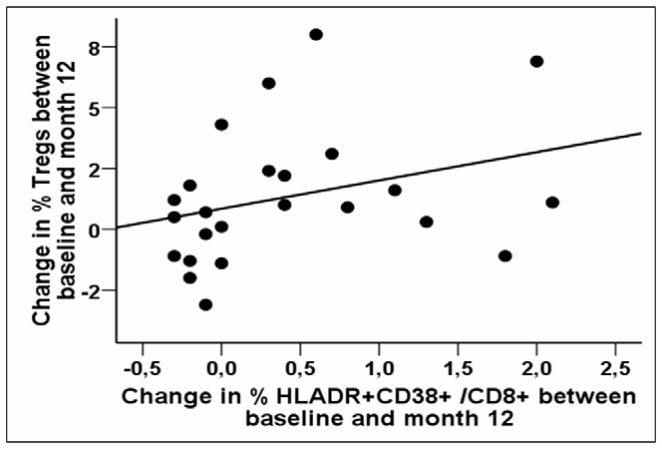
The increase in the proportion of Tregs between baseline and M12 is independently related to the extent of generalized T-cell activation following treatment interruption. Relationship between the change in the percentages of CD8+ T cells co-expressing HLA-DR and CD38 (x-axis) and the changes in the proportion of CD25+ CD127−/low Tregs among CD4+ T cells (y-axis) between baseline (prior to TI) and M12 of TI. Spearman's correlation coefficient R = 0.433; p = 0.031.

We looked for the factor(s) that influence Treg evolution between baseline and M12 of TI in multivariable analyses, and found that the only factor independently related to the increase in Tregs'proportion following TI was the increase in the proportion of CD8^+^T cells co-expressing HLA-DR and CD38 (n = 25, β = 3.345±1.025, p = 0.005) (Fig. 3).

### In patients with viral replication following TI, the proportion of Tregs did not negatively correlate with the level of CD8 T-cell activation

Interestingly, contrary to baseline, we no longer found, at M12 of TI, a negative correlation between the proportion of CD25^+^CD127^−/low^Tregs and the percentage of CD8^+^T cells expressing HLA-DR (Fig. 1b). We even found a positive relationship between the proportion of Tregs and the proportion of CD8^+^ T cells and CD4^+^ T cells expressing CD38 (n = 25, r = 0.539; p = 0.010 and r = 0.616; p = 0.004, respectively). These data suggest that, following TI, Tregs are no longer capable of controlling immune activation resulting from viral replication.

### Tregs retain a suppressive activity in patients undergoing cART Interruption

In order to assess if the Tregs detected after TI retain a suppressive activity, we compared the proliferative capacity of total CD4^+^T cells and of CD4^+^T cells depleted of CD4^+^CD25^+^T cells in a subgroup of 10 patients for whom fresh PBMC were available. Depletion of Tregs-containing CD4^+^CD25^+^cells led to an increase (≥2-fold) in lymphoproliferative responses (LPR) to HIV-p24 and to CMV antigen in 4/10 patients and 5/10 patients, respectively. Altogether, depletion of Tregs-containing CD4^+^CD25^+^cells led to an increase of LPR to at least one antigen (HIV-p24, CMV or PPD) in 7/10 patients, indicating that CD4^+^CD25^+^T cells from patients who stopped ART retain a suppressive activity.

### Treg cells at baseline did not affect the immunovirologic outcome at 12 months of cART interruption

We then investigated whether the proportion and/or the numbers of CD25^+^CD127^−/low^Tregs at baseline may independently influence the extent of the CD4 cell decline and the degree of virologic rebound. In addition, we sought other immunologic and virologic parameters at baseline that may independently predict the CD4 T-cell drop and the increase in viral load following TI.

We did not find any relationship between the proportion or the number of Tregs at baseline and the immunovirologic outcome at M12. Equally, we did not find any impact of HIV-specific CD4 and CD8 T-cell responses before TI on the immunological and virologic status achieved at M12 of TI.

**Figure 4:**
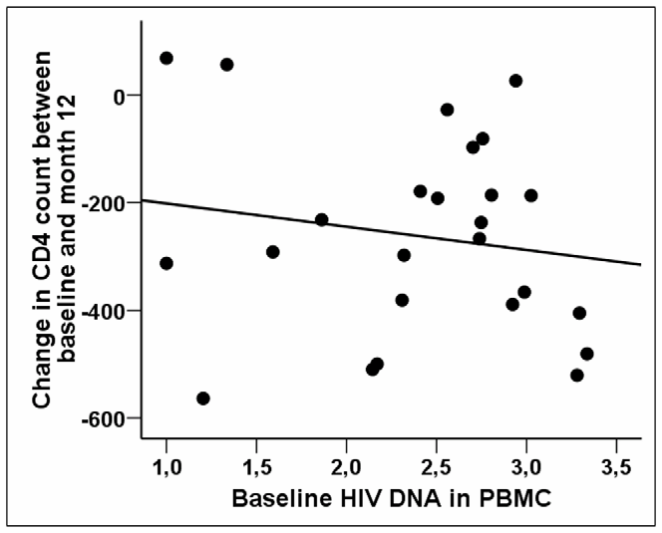
Predictors of the immunovirologic outcome at month 12 of treatment interruption. Baseline HIV-DNA levels predict the CD4 T-cell decline between baseline and M12 of TI. Relationship between the levels of HIV-DNA in PBMCs at baseline prior to TI (x-axis) and the change in CD4 cell counts between baseline and M12 of TI (y-axis). Spearman's correlation coefficient R = −0.578; p = 0.002.

The factors that independently predicted the CD4 T-cell decline between baseline and M12 were the CD4 T-cell count and the HIV-DNA levels in PBMCs at the time of TI (n = 25, β = −79±16, p<0.001 and β = −127±41, p = 0.006, respectively). The decline of CD4 T-cell count was found to be more substantial whenever HIV-DNA levels or CD4 T-cell counts were high. Fig. 4 illustrates the relationship between baseline HIV-DNA levels and the extent of CD4 cell decline between baseline and M12.

**Figure 5:**
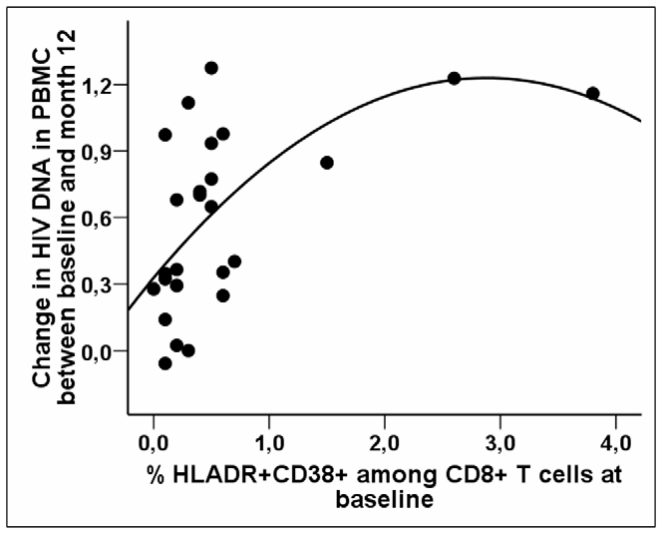
The proportion of CD8+ T cells co-expressing HLA-DR and CD38 at baseline predicts the increase in HIV-DNA levels between baseline and M12 of TI. Relationship between the percentages of CD8+ T cells co-expressing HLA-DR and CD38 at baseline prior to TI (x-axis) and the change in HIV-DNA levels in PBMCs between baseline and M12 of TI (y-axis). The line corresponds to the polynomial regression line (order 2), the overall non parametric Spearman's correlation coefficient R is estimated as 0.552; p = 0.004, while it is 0.440 (p = 0.0357) after removing the two most extreme values.

The increase in HIV-DNA between baseline and M12 was independently predicted by the number of CD4^+^ T cells and the proportion of CD8^+^ T cells co-expressing CD38 and HLA-DR at baseline (n = 25, β = 0.098±0.034; p = 0.008 and β = 0.231±0.073; p = 0.004, respectively). The increase in HIV-DNA level was more important when CD4 T-cell counts and immune activation were high. The relationship between the proportion of CD8^+^ cells co-expressing HLA-DR and CD38 and the increase in HIV-DNA levels between baseline and M12 is shown in Fig. 5.

Finally, the HIV-DNA level in PBMCs and the proportion of activated CD38^+^HLA-DR^+^CD8^+^ T cells before TI were independent predictors of the level of plasma HIV-RNA achieved at M12 (n = 25, β = 0.790±0.100; p<0.001 and β = 0.172±0.082; p = 0.048, respectively).

## Discussion

Data from the present study strongly suggest that regulatory T cells are capable of controlling residual immune activation in patients under cART but not the immune activation resulting from viral replication after cART interruption.

Patients included in the present study were treated early in the course of HIV infection and exhibited, at baseline under cART, high CD4 cell counts, undetectable plasma HIV-RNA and relatively low levels of HIV-DNA in PBMCs [30]. At baseline we found an inverse relationship between HIV-DNA levels and CD4 T-cell counts. The level of HIV-DNA has been reported to be a major and independent predictor of disease progression in untreated patients with primary HIV infection [30], [32], [33]. It has been suggested that the level of HIV-DNA in PBMCs reflects the number of circulating HIV-infected cells representing the cellular stock.

Despite the limited size of the patient population and the fact that we only studied the blood compartment, our study is important since we had the opportunity to investigate Tregs when viral replication starts again after a long period of viral control under effective cART.

As expected, following TI plasma HIV-RNA and HIV-DNA levels in PBMCs increased while CD4 cell counts decreased and circulating CD4 and CD8 T cells became activated. Thus, the proportion of CD4^+^ and CD8^+^ T cells expressing CD38 was found to significantly increase unlike T cells expressing HLA-DR. We found, in our study, a relatively low proportion of HLA-DR^+^ T cells because we considered, as positive cells, only T cells which expressed high levels of HLA-DR. Nevertheless, some discrepancies between the expression of HLA-DR and CD38 markers on activated T cells have already been reported. HLA-DR expression was found to correlate with Ki-67 expression and but not consistently with viral load in HIV-infected patients [34], [35]. Thus, HLA-DR^+^ CD38^−^ T cells probably represent proliferating cells whereas CD38^+^ T cells preferentially increase as a result of viral replication [36]. Finally, none of the available cellular markers of T-cell activation or proliferation appears to be able alone to consistently and robustly assess the level of immune activation across all subsets of HIV-infected patients [37]. Thus, we chose to evaluate immune activation by the proportion of CD4 and CD8 T cells expressing HLA-DR or CD38 or co-expressing both markers.

At baseline, the inverse relationship between the proportion of CD4^+^CD25^+^CD127^−/low^Tregs and the proportion of activated HLA-DR^+^CD8^+^T cells strongly suggests that *in vivo*, in patients with cART-mediated viral suppression, Tregs are capable of controlling residual immune activation. However, as we did not find a negative relationship between the proportion of CD8 T cells expressing CD38 and the proportion of Tregs, it cannot be excluded that Tregs control spontaneous T-cell proliferation rather than generalized immune activation in this context. The recent report of a preservation of Tregs in peripheral blood of elite suppressors also suggests that Tregs are beneficial rather than damaging in HIV-infected patients controlling HIV replication [23]. However, the inverse relationship between the proportion of peripheral Tregs and specific anti-HIV CD8 T-cell responses that we found, indicated that, as demonstrated *in vitro* [9], [10], [38], Tregs can suppress HIV-specific CD8 T-cell responses *in vivo*. The positive relationship between HIV-DNA levels and the proportion of peripheral Tregs may indicate that Tregs are driven directly by HIV, as suggested by previous reports of HIV-specific Tregs in patients with chronic or primary infection [8], [39] and/or indirectly by immune activation resulting from HIV replication.

To gain insight into the role of Tregs in vivo, we further analysed Treg evolution following cART interruption. We found, in the multivariable analysis, that the increase in the proportion of Tregs between baseline and M12, although quite modest, was only independently related to the level of generalized immune activation (i.e. the proportion of CD8^+^T cells co-expressing CD38 and HLA-DR). This indicated that the proportion of Tregs among CD4^+^ T cells expanded as a result of immune activation. Viral replication and immune activation following TI increase interactions between HIV or envelope proteins and CD4 or HIV-co-receptors, and may favor the peripheral conversion of memory activated CD4 T cells into Treg [40]. In agreement with recent results [23], we found no evidence for a preferential depletion of Tregs in viremic patients off-treatment.

CD4 and CD8 T-cell activation at M12 of TI positively correlated with plasma VL suggesting that, in patients interrupting cART, immune activation is primarily driven by HIV. Interestingly, we did not observe after M12 of TI an inverse relationship between the proportion of Tregs and the proportion of CD8^+^T cells expressing HLA-DR and/or CD38. We found a positive relationship between the proportion of Tregs and the proportion of CD4^+^ T cells expressing CD38 suggesting that, as previously suggested in viremic patients [41], CD4^+^CD25^+^CD127^lo^ may include activated CD4^+^ T cells and/or that CD4 T cell activation drives Treg expansion. The lack of inverse relationship between the proportion of Tregs and CD8 T-cell activation following TI strongly suggests that, in the situation of viral replication and subsequently increased immune activation, Tregs are no longer capable of regulating T-cell activation in most patients, as recently described in ART-naive patients [22]. The ability of Tregs to control immune activation *in vivo* thus may vary according to the level of T-cell activation needed to be contained; this may explain conflicting results previously reported. Thus, in very early stage of SIV infection in African green monkeys, when immune activation is low despite high viral load, Tregs might be able to control immune activation [20].

The inefficiency of Tregs to control immune activation in the context of TI may result from the decrease in the absolute numbers of Tregs and/or an insufficient increase in the proportion of Tregs or from a defect in Treg suppressive function. Finally, Tregs could have migrated to lymphoid tissues, as suggested by previous studies in humans and macaques [17], [18], [42]. We previously demonstrated that CD4^+^CD25^+^Tregs from cART-treated chronically HIV-infected patients exerted a suppressive activity on CD4 T-cell proliferative responses against CMV and HIV-p24 [8]. Accordingly to recent published data [13], [23], we found that, even in patients with detectable viral load, the Tregs-containing CD4^+^ CD25^+^ T-cell population from peripheral blood retains a suppressive function on CMV and HIV-specific proliferative responses.

Following cART interruption, the proportion of Tregs increased as a consequence of T-cell activation driven by HIV replication; anti-HIV specific CD8 T-cell responses enhanced and broadened. Previous reports had suggested a role for the intensity and the breadth of memory Gag-specific CTL on viral control [43]. We found that HIV-DNA levels at M12 negatively correlated with anti-gag but not total anti-HIV CD8 T-cell responses at M12. A lack of correlation between the increase in HIV-specific CD8^+^ T lymphocyte frequencies and the viral set point after TI has already been observed [31]. The polyfunctional profile of virus-specific CD8 T-cells seems to be a better correlate of the virologic outcome in patients who had interrupted cART [44].

We sought for factors at baseline which may independently predict the CD4 T-cell drop and the increase in viral load following TI and found no impact of Tregs on the immunovirologic outcome at 12 months of TI. It is commonly accepted that persistent immune activation plays a central role in driving CD4 T-cell depletion [1], [45], [46]. Our results indicate that the level of HIV-DNA in PBMCs reflecting, at least in part, the size of the HIV reservoir and the number of CD4^+^ T cells, preferential targets of the virus, were independent predictors of CD4 T-cell decline. We did not find any independent relationship between CD4 T-cell decline and the level of CD4 and CD8 T-cell activation at least at baseline. In contrast, we found that the increase in HIV-DNA levels in PBMCs between baseline and M12 was independently predicted by the number of CD4^+^ T cells and the level of generalized T-cell activation at baseline. The latter finding suggests that T-cell activation may directly impact the HIV reservoir.

Finally, the HIV-DNA level in PBMCs before TI was the strongest independent predictor of the level of plasma HIV-RNA achieved at M12. In the main trial, we found that HIV-DNA level at treatment interruption, in addition to CD4 nadir and prior AIDS event, was the only significant risk factors of reaching criteria to resume cART [47].

In summary, our data indicate that in patients interrupting cART, the level of HIV-DNA in PBMCs before TI independently predicts the extent of CD4 T-cell decline and of viral control achieved at M12, the latter being also predicted by the degree of generalized immune activation before TI. Tregs seem to be efficient in controlling residual immune activation in patients with viral suppression under cART. However following TI, even if Treg cells maintained a suppressive function, the inadequate increase in the proportion of Tregs and/or the decrease in the absolute number of Tregs resulted in a failure to control immune activation. Further studies are needed to better define the impact of different types of regulatory T cells on the natural history of HIV infection from the primary infection.

## Supporting Information

Checklist S1 CONSORT Checklist.
(0.02 MB PDF)

Protocol S1 Trial protocol.
(0.02 MB PDF)

Flowchart S1 (0.03 MB DOC)
