# Supplementary material for: Relationship between Regulatory T Cells and Immune Activation in Human Immunodeficiency Virus-Infected Patients Interrupting Antiretroviral Therapy
Source: PLoS One. 2010 Jul 21;5(7):e11659. doi: 10.1371/journal.pone.0011659 (PMC2908121; doi:10.1371/journal.pone.0011659)
Supplement: Checklist S1 — CONSORT Checklist. (0.02 MB PDF) [file pone.0011659.s001.pdf]

ClinicalTrials.gov
Protocol Registration System

Protocol Registration Receipt
2005-07-01
IND Grantor: IND Number: IND Serial Number:

Long term supervised treatment interruption in HIV-infected patients started on treatment with CD4 over 350/mm3 and plasma HIV RNA below 50 000/mL.

This study is no longer recruiting patients.

**Sponsored by:**	French National Agency for Research on AIDS and Viral Hepatitis
**Information provided by:**	French National Agency for Research on AIDS and Viral Hepatitis

Purpose

This trial is aimed at studying the safety of long term supervised treatment interruption in HIV infected patients with CD4 over 350/mm3 and plasma HIV RNA under 50 000/mL. Another aim of this study is to assess the immunological and virological factors associated with the duration of treatment interruption.

Condition	Treatment or Intervention	Phase
HIV Infections	Procedure: Treatment interruption	Phase 3

Study Type: Interventional
Study Design: Treatment, Non-Randomized, Open Label, Uncontrolled, Single Group Assignment, Efficacy Study

Official Title: Long term supervised treatment interruption in HIV-infected patients who started antiretroviral treatment with CD4 over 350/mm3 and plasma HIV RNA below 50 000/mL. ANRS 116 trial SALTO

Further Study Details:

Primary Outcomes: Proportion of patients who did not resume antiretroviral treatment at 12 months
Secondary Outcomes: Time to resume antiretroviral treatment with CD4 cell count equal or below 300/mm3; Proportion of patients who did not resume antiretroviral treatment at M 24 and M 36; Predictive factors associated with the time of restart of antiretroviral therapy:; Proviral HIV DNA at baseline and during follow-up; Plasma HIV RNA at baseline and during follow-up; CD4 T cell and CD8 T cell HIV specific responses at baseline and after 12 months; Change in lipodystrophy clinical score and quality of life during the follow-up; Criteria to resume antiretroviral treatment: CD4T cell count below or equal to 300/mm3; The occurrence of an

AIDS defining event

Expected Total Enrollment: 130

Study Start: 2003-02

The limitations of the drugs used against HIV include their toxicity, their tolerability, their propensity to induce resistance when not taken with absolute regularity and their cost. Treatment interruption in patients receiving antiretroviral treatment in the setting of chronic infection are associated with viral rebound and rapid CD4 T cell decrease conducting to antiretroviral therapy restart. In patients with high CD4+ cell counts (patients receiving treatment of chronic infection with controlled viremia and patients who are receiving HAART now in whom treatment would not have been started based on current guidelines), we evaluated the safety of long term supervised treatment interruption. Another aim of this study was to assess the immunological and virological factors associated with the duration of treatment interruption (proviral HIV DNA at baseline and during follow-up, plasma HIV RNA at baseline and during follow-up, CD4 T cell and CD8 T cell HIV specific responses at baseline and after 12 months).

Eligibility

Ages Eligible for Study: 18 Years - N/A, Genders Eligible for Study: Both

Criteria

Inclusion Criteria: - Males and non pregnant females - 18 years of age and older - Who have confirmed laboratory diagnosis of HIV infection - Started on first line antiretroviral treatment with CD4 over 350/mm3 and plasma HIV RNA below 50 000/mL -Ongoing Antiretroviral therapy at inclusion with CD4 over 450/mm3 and plasma HIV RNA below 5000/mL

Exclusion Criteria: - HBV-HIV co-infection receiving lamivudine therapy - Ongoing immunotherapy including IL2, interferon or HIV specific vaccine - Pregnancy or project of pregnancy

Location and Contact Information

France
Service d'immunologie Clinique, Höpital Européen Georges Pompidou, Paris, 75015, France

Study chairs or principal investigators

Christophe Piketti, MD, Principal Investigator
Hopital Georges Pompidou Paris France
Dominique Costagliola, Study Chair
Inserm U720

More Information

Study ID Numbers ANRS 116 SALTO
Record last reviewed 2005-07
Health Authority: France: Afssaps - French Health Products Safety Agency
