# Supplementary material for: Relationship between Regulatory T Cells and Immune Activation in Human Immunodeficiency Virus-Infected Patients Interrupting Antiretroviral Therapy
Source: PLoS One. 2010 Jul 21;5(7):e11659. doi: 10.1371/journal.pone.0011659 (PMC2908121; doi:10.1371/journal.pone.0011659)
Supplement: Flowchart S1 — (0.03 MB DOC) [file pone.0011659.s003.doc]

**Follow-up**

**Analysis**

Assessed for eligibility **(n=119)**

**Enrollment**

**Lost to follow-up** (n=4, month 17, month 24 (2 patients), and month 27)

**Discontinued** **for pregnancy** (n=4, months 2, 21, 24 and 32)

**Died** (n=1, month 21) of a ruptured cerebral aneurysm

**Analysed** (n=116)

Patients included in the sub-study (**n=25**)

None of the 25 patients had resumed cART at month 12

Excluded **(n=3)**

Refused to participate (n=3)
